# Upgrade Rate of Ductal Carcinoma In Situ to Invasive Carcinoma and the Clinicopathological Factors Predicting the Upgrade Following a Mastectomy: A Retrospective Study

**DOI:** 10.7759/cureus.35735

**Published:** 2023-03-03

**Authors:** Zaid Al-Ishaq, Hamed Hajiesmaeili, Ehsanur Rahman, Muskaan Khosla, Tapan Sircar

**Affiliations:** 1 Surgical Oncology, Sultan Qaboos Comprehensive Cancer Care and Research Centre, Oman, GBR; 2 Breast Surgery, Royal Wolverhampton NHS Trust, Wolverhampton, GBR

**Keywords:** breast cancer, upgrade, sentinel lymph node biopsy, mastectomy, multifocality, invasive ductal breast carcinoma, dcis, breast cancer biology

## Abstract

Background

The rate of upgrading ductal carcinoma in situ (DCIS) to invasive cancer varies widely in the literature with no consensus regarding sentinel lymph node biopsy (SLNB) for DCIS; however, some guidelines do recommend it in the event of a mastectomy. The primary aim of this study was to determine the upgrade rate of DCIS to invasive carcinoma (IC) in patients undergoing mastectomy for DCIS and identify the clinicopathological predicting factors for the upgrade. The secondary aim was to determine the SLNB positivity rate.

Methodology

We retrospectively analysed consecutive patients with DCIS diagnosed through a biopsy who then underwent mastectomy over a 10-year period (2010 to 2020). Clinical, radiological, and histological variables were collected from medical records.

Results

We studied 143 women (mean age = 57.4 years, range = 26-85 years) who underwent mastectomy for DCIS identified on biopsy. Almost two-thirds (62.9%, 90/143) of the patients were detected on screening mammography, while 35.6% (51/143) were diagnosed following presentation with either an area of palpable concern or nipple discharge. The most common mammographic presentation of DCIS was calcification (83.9%, 120/143), and, in 85.9% of the patients, the mammographic lesion was more than 20 mm. High-grade DCIS was noted in 76.9% of preoperative biopsy results, while the rest was either low or intermediate-grade DCIS. Overall, 24.5% (35/143) were upgraded to IC (upgraded group) on postoperative histology, whereas 108/143 remained DCIS postoperatively (pure DCIS group). The positivity rate of SLNB was 4.8%. Multifocality was the only significant predictor of IC on multivariate analyses of clinicopathological predictors (odds ratio = 3.0, 95% confidence interval = 1.0-8.7). The presence of comedonecrosis was higher in the upgraded group compared to the pure DCIS group (42.9% vs. 27.8%), but this was not statistically significant.

Conclusions

In our study cohort, nearly one in four (24.5%) patients were upgraded from DCIS to IC on postoperative histology, with an SLNB positivity rate of 4.8%. This is important when counselling patients regarding the risk of coincident occult IC and the importance of SLNB at the time of mastectomy. Multifocality on preoperative imaging was the only significant predictive factor. Based on this result, we recommend that SLNB should also be considered if patients have multifocal DCIS and planned for oncoplastic breast-conserving surgery. However, further studies are required to investigate the association between multifocal DCIS and the risk of upgrading to IC.

## Introduction

Ductal carcinoma in situ (DCIS) is a common form of non-invasive breast cancer. More than 20% of breast cancers diagnosed by screening mammography are DCIS [1]. In the United Kingdom, around 6,900 patients are diagnosed with DCIS every year, most of whom are asymptomatic and are usually picked up from the screening program [2]. As almost all patients undergo surgical excision, the natural history of DCIS is poorly understood; however, there is a growing understanding that DCIS is a heterogeneous disease process [3]. As cancer cells in DCIS do not spread beyond the basal cell layer in the mammary duct, theoretically, a sentinel lymph node biopsy (SLNB) is not essential for patients with DCIS.

Pathological diagnosis using core needle biopsy or vacuum-assisted core biopsy cannot completely exclude invasive components before surgery [4,5], which, if found, will upgrade the DCIS to invasive carcinoma (IC). This will lead to the need for the staging of the disease through axillary lymph node evaluation. Therefore, the National Institute for Health and Care Excellence (NICE) guidelines suggest that all patients who undergo a mastectomy for DCIS should be offered an SLNB [6]. The rationale is that the potential finding of occult invasive cancer in the mastectomy specimen which would subsequently warrant axillary staging and SLNB is technically challenging after mastectomy, and, hence, the patient would have to undergo either axillary sampling or clearance with all the potential morbidities of these procedures.

A French multi-centre study (Cinnamome) by Tunon-de-Lara et al. demonstrated a 39% upgrade rate of DCIS to micro or invasive cancer following mastectomy, and having performed a successful SLNB, 67% of these patients avoided needing axillary lymph node clearance [7].

Interestingly, another study by Watanabe et al. suggested that routine SLNB in patients with mastectomy for DCIS is overtreatment and should only be reserved for patients at a high risk of upgrading to IC such as a palpable mass, ultrasound findings classified as category 4 or 5, or widely distributed non-mass abnormality. This was due to a very low prevalence of sentinel lymph node metastasis in their cohort of patients [8].

There have been some studies that have investigated factors that can help predict the upgrade of DCIS to IC on postoperative histology. Price et al. suggested predicting factors such as clinically palpable lesions, a large extent of disease on imaging, a mass on preoperative imaging, multifocality, and multicentricity [9]. Another study by Tunon-de-Lara et al. found that expression of the human epidermal growth factor receptor 2 (HER-2) receptor was an independent predictor of invasive disease, and high nuclear grade was associated with an increased risk of micro-invasion and invasion [7].

In this retrospective, single-centre study, we examined consecutive patients who underwent a mastectomy for DCIS between January 1, 2010, and December 31, 2020, investigating primarily the rate of upgrade of DCIS to IC and the possible clinicopathological predictors of the upgrade. The secondary aim was to assess the SLNB positivity rate.

## Materials and methods

We retrospectively reviewed 143 patients with a pre-mastectomy diagnosis of only DCIS without evidence of micro-invasion either on core or excision biopsy over a period of 10 years between January 1, 2010, and December 31, 2020, from a single institute. We collected demographic, clinical, radiological, and histological data from medical records and clinical databases. Patients whose pre-mastectomy pathology report was indicative of DCIS and who displayed an invasive component in the final pathological report were considered as upgraded DCIS. Upgraded DCIS was defined as showing IC in the pathological report of the final mastectomy specimen (detected by basement membrane invasion).

Descriptive analysis was performed to compare the demographic features, clinicopathological features, and breast imaging findings between DCIS and upgraded invasive groups. The chi-square test or Fisher’s exact test were used when appropriate. Continuous data were reported as mean and range, and categorical data were reported as counts and percentages.

Independent predictors of IC were analysed using logistic regression analysis. A stepwise selection method with both forward and backward elimination was used to select variables for the multivariate logistic regression model. All statistical analyses were performed with R version 4.0.3. (R Foundation for Statistical Computing, Vienna, Austria). A p-value <0.05 was considered statistically significant.

This study was approved by the Institutional Review Board of the institution. The need for written informed consent was waived due to the retrospective nature of the study.

## Results

The clinicopathological characteristics of 143 patients with a preoperative histopathologic diagnosis of DCIS are listed in Table 1. The mean age was 57.4 years (range = 26-85 years). These characteristics were compared between patients diagnosed with only DCIS (pure DCIS group) and those upgraded to IC (upgraded group). In our cohort, 108 out of 143 (75.5%) patients with a preoperative diagnosis of DCIS were confirmed to remain as DCIS, while 25 out of 143 (24.5%) patients were upgraded to IC. Factors such as mammogram findings (p = 0.01) and multifocality (p = 0.027) varied significantly between the two groups (Table 1).

**Table 1:** Demographics, clinicopathological features, and imaging findings of the DCIS-only group and the upgraded DCIS with the invasive component group after mastectomy. DCIS = ductal carcinoma in situ; IC = invasive carcinoma

	Total cases, 143 (100%)	Pure DCIS group, 108 (75.5%)	Upgraded to IC group, 35 (24.4%)	P-value
Age category (years)				0.752
≤50	46 (32.1%)	36 (33.3%)	10 (28.6%)	
>50	97 (67.8)	72 (66.7%)	25 (71.4%)	
Ultrasound findings				0.330
Calcification	16 (11.1%)	11 (10.2%)	5 (14.3%)	
Cysts/BBC	14 (9.7%)	12 (11.1%)	2 (5.71%)	
Mass lesion	47 (32.8 %)	32 (29.6%)	15 (42.9%)	
Normal	42 (29.3%)	31 (28.7%)	11 (31.4%)	
Not done	19 (13.2%)	17 (15.7%)	2 (5.71%)	
Prominent ductal pattern	5 (3.4%)	5 (4.63%)	0 (0.00%)	
Size on the mammogram (mm)				0.398
≤20	18 (13.9%)	15 (15.8%)	3 (8.82%)	
>20	111 (86%)	80 (84.2%)	31 (91.2%)	
Mammogram finding				0.024
Asymmetric density	13 (9%)	8 (7.41%)	5 (14.3%)	
Microcalcification	121 (84.6%)	93 (86.1%)	28 (80.0%)	
Mass lesion	2 (1.3%)	0 (0.00%)	2 (5.71%)	
Normal	7 (4.8%)	7 (6.48%)	0 (0.00%)	
Multifocal				0.027
No	121 (84.6%)	96 (88.9%)	25 (71.4%)	
Yes	22 (15.3%)	12 (11.1%)	10 (28.6%)	
Presentation				0.609
Incidental (symmetrizing breast reduction)	2 (1.3%)	2 (1.85%)	0 (0.00%)	
Screening	90 (62.9%)	70 (64.8%)	20 (57.1%)	
Symptomatic	51 (35.6%)	36 (33.3%)	15 (42.9%)	
Nuclear grade				0.467
Low-intermediate	33 (23%)	27 (25.0%)	6 (17.1%)	
High	110 (76.9%)	81 (75.0%)	29 (82.9%)	
Comedonecrosis				0.144
No	98 (68.5%)	78 (72.2%)	20 (57.1%)	
Yes	45 (31.4%)	30 (27.8%)	15 (42.9%)	

We investigated the clinicopathological factors that could predict IC in the surgical specimen obtained during the operation. The results revealed that multifocality of the lesion (odds ratio (OR) = 3.5, confidence interval (CI) = 1.3-9.5, p = 0.012 in univariate analysis; OR = 3.0, CI = 1.0-8.7, p = 0.043 in multivariate analysis) was the only significant risk factor for IC in both univariate and multivariate analyses of clinicopathological predictors (Table 2).

**Table 2:** Clinicopathological predictors of IC in patients with a preoperative diagnosis of DCIS. DCIS = ductal carcinoma in situ; IC = invasive carcinoma; OR = odds ratio; CI = confidence interval

Variable	Risk for invasive carcinoma			
	Univariate analysis		Multivariate analysis	
	OR (95% CI)	P-value	Adjusted OR (95% CI)	P-value
Age				
>50	1.411 (0.59-3.38)	0.439		
Ultrasound findings				
Mass lesion	1.06 (0.31-3.62)	0.92		
Size on the mammogram				
>20 mm	1.94 (0.52-7.16)	0.321		
Mammogram findings				
Microcalcification	0.5 (0.15-1.65)	0.252	0.58 (0.169-2.03)	0.397
Multifocality				
Yes	3.54 (1.32-9.5)	0.012	3.011 (1.037-8.74)	0.043
Presentation				
Symptomatic	1.67 (0.74-3.78)	0.214		
Grade				
High	1.17 (0.42-3.22)	0.766		
Necrosis				
Yes	1.71 (0.77-3.82)	0.19	2.037 (0.874-4.75)	0.099

Although the presence of comedonecrosis was higher in the upgraded group compared to the pure DCIS group (42.9% vs. 27.8%), this was not statistically significant.

Other factors such as age, ultrasound findings, the size and type of mammographic abnormality, the route of presentation, and nuclear grade were not significant predictors of IC.

In the upgraded group (n = 35), most patients (94.4%, n = 33) had IC of no special type, mainly grade 2 (45.7%, n = 16). Invasive lobular carcinoma was found in only two (5.7%) cases.

SLNB was performed for 86% (n = 123/143) of patients at the time of mastectomy. Out of 123 patients, six had positive SLNB (4.8%). However, when we examined the upgraded group (35 patients), the SLNB was positive in six (17.1%) patients. The average number of sentinel lymph nodes removed was three (range = 2-5 lymph nodes). Three patients then underwent completion axillary lymph node clearance with no further involved lymph node, while the remaining three patients underwent regional radiotherapy.

## Discussion

Our study assessed the rate of upgrading DCIS to IC and the SLNB positivity in patients who underwent mastectomy for DCIS. The clinicopathological predictors of this upgrade were also investigated. The upgrade rate to IC was found to be 24.5% (n = 35/143 patients). Our result (24.5%) was consistent with those of previous studies [1,10,11]. However, some studies have reported a higher rate. Tunon-de-Lara et al. in their multi-centre study reported a 39% risk of upgrade to IC [7] while Jisun et al. reported a 42.7% rate [12]. This variation can be related to the heterogeneity of DCIS and the pathologist’s interpretation of factors [10,13].

Due to the consequent risk of axillary lymph node involvement [14], upgrading DCIS to IC carries a significant impact on patients and surgeons.

To date, the indication of SLNB in DCIS cases undergoing mastectomy remains controversial. The National Comprehensive Cancer Network and NICE guidelines recommend SLNB for DCIS patients in the event of mastectomy while Dutch guidelines recommend SLNB for patients with a high risk of invasion regardless of the type of surgical treatment (mastectomy or breast-conserving surgery). A high risk of invasion was defined as a tumour size >2.5 cm, palpable lesion, grade 3 DCIS, extensive calcifications and age <55 years [6,11,15]. However, Chehade et al. in their meta-analysis of 48 studies on SLNB in DCIS have identified high-grade DCIS >20 mm as the only factor to perform SLNB [16]. In our institution, we follow the NICE guidelines and 86% of our patients underwent SLNB following mastectomy for DCIS. Overall, 14% (20/143) of patients did not undergo SLNB due to previous axillary staging (five patients), patients offered but declined the procedure (three patients), and in 12 patients no reason was documented in medical records.

The SLNB positivity rate in our study was 4.8% (6/123 patients) which is in agreement with other studies [11,12,17], and a recent meta-analysis by Davey et al. analysed the data from 4,388 patients in 16 studies and found a rate of 4.9% and recommended further well-designed randomised control trials to fully establish the necessity of SLNB for patients diagnosed with DCIS [14].

Our study also investigated the factors associated with upgrading DCIS to IC. There is no consensus in the literature regarding the predictors of upgrading pure DCIS to IC, though previous studies have reported young age, symptomatic presentation, specimen number, the extent of microcalcification, the size of the mass, high nuclear grade, axillary lymph node involvement, multi-centric lesion, contralateral lesion, and the presence of HER-2 overexpression as predictors of DCIS with IC prior to surgery [10,12,18-21]. Due to the small sample size and inconsistent results, the authors concluded that no predictive factors for IC were available clinically [4].

Brennan et al. in their meta-analysis could not find a specific median age associated with upgrading, although they reported that patients less than 52.5 years of age were relatively associated with a higher risk of upgrading than the older age groups [10]. This is consistent with our result where age was not a predictor of upgrade.

In our study, the ultrasound scan finding of the mass lesion was not a significant predictor of IC. This finding is not in agreement with other studies which highlighted that mass formations on ultrasound were a significant predictor of IC [1,22]. This may be due to the under-representation of this feature in our cohort owing to the small sample size of our study.

In our cohort, the type of mammographic finding and mammographic lesions measuring more than 20 mm were not found to be significant predictive factors. In contrast, Brennan et al. in their meta-analysis reported that lesions measuring more than 20 mm at the time of imaging and the presence of a mammographic mass associated with mammographic microcalcification were strongly associated with the upgrade to IC [10]. Our findings could be explained by the small number of patients who had mammographic findings of mass lesions (1.4%).

While symptomatic presentation (palpable lump) on clinical examination has been reported to increase the risk of invasive disease [23]. In our study, we could not confirm that as most cases (62.9%) in our cohort were detected early through the breast screening programme and were clinically not palpable.

The high nuclear grade was not a significant predictor of IC in our cohort. This finding was consistent with several studies which reported no association between the high grade and the likelihood of upstaging [4,23-26] However, other studies [4,10,27] found that the high nuclear grade was a predictor of IC. This discrepancy between the studies explained by some authors is a reflection of the controversy excluding micro-invasion from histologic grade with the recommendation to investigate this factor in future studies [4].

The type of DCIS, such as non-cribriform type, papillary type, solid type, and comedonecrosis, have been reported to predict upgrading to invasive cancer [28]. In our study, we reviewed the presence of the comedonecrosis subtype and found that the presence of comedonecrosis was higher in the upgraded group compared to the pure DCIS group (42.9% vs. 27.8%), but this was not statistically significant.

Though Lee et al. reported no statistical significance of multifocality in predicting IC [4], in our study, the presence of a multifocal lesion on the mammogram was the only significant predictor of IC in both the univariate and multivariate logistic regression (p = 0.012 and 0.043, respectively).

Based on our results and with the increasing trend toward breast-conserving surgery and extreme oncoplastic surgeries for patients with multifocal disease, we feel that SLNB should be considered in the above patients. This will avoid a second operation and a low identification rate of SLN following oncoplastic breast surgery. However, further studies are required to investigate the association of multifocal DCIS with the risk of upgrading to IC.

Our study had some limitations. It was a retrospective study conducted with a relatively small cohort, as we excluded patients with a preoperative diagnosis of micro-invasion or invasive foci to get the real upgrade rate. Furthermore, we did not include other predictive factors such as hormone receptors or HER-2 receptor status as it is not in our routine practice to look for them in patients with pure DCIS.

## Conclusions

The upgrade rate of DCIS to IC following a mastectomy was seen in one in four (24.5%) patients while the sentinel lymph node positivity rate was 4.8%. These results will help in preoperative counselling and informed consent for patients having a mastectomy for DCIS. The presence of imaging findings of multifocality was associated with a higher chance of upgrading to IC. Hence, the option of SLNB should also be considered in patients undergoing breast-conserving surgery, especially oncoplastic surgery for multifocal DCIS. However, further studies are required to investigate the association between multifocal DCIS and the risk of upgrading to IC.
